# Identification of urine neutrophil gelatinase-associated lipocalin molecular forms and their association with different urinary diseases in cats

**DOI:** 10.1186/s12917-019-2048-9

**Published:** 2019-08-27

**Authors:** Po-Han Wu, Wei-Li Hsu, Pei-Shiue Jason Tsai, Vin-Cent Wu, Han-Ju Tsai, Ya-Jane Lee

**Affiliations:** 1 Institute of Veterinary Clinical Science, School of Veterinary Medicine, College of Bio-Resources and Agriculture, National Taiwan University, No. 153, Sec. 3, Keelung Rd, Taipei, 106 Taiwan; 2 Graduate Institute of Microbiology and Public Health, College of Veterinary Medicine, National Chung-Hsing University, 145 Xingda Rd., Taichung, 40227 Taiwan; 3 Department of Veterinary Medicine, School of Veterinary Medicine, National Taiwan University, No.1, Sec.4, Roosevelt Rd, Taipei, 106 Taiwan; 4 National Taiwan University Hospital, No.1, Changde St., Zhongzheng Dist., Taipei, 10048 Taiwan; 5 National Taiwan University Veterinary Hospital, College of Bio-Resources and Agriculture, National Taiwan University, No. 153, Sec. 3, Keelung Rd, Taipei, 106 Taiwan

**Keywords:** Molecular forms, Biomarker, NGAL, MMP-9, Feline urinary diseases

## Abstract

**Background:**

Neutrophil gelatinase-associated lipocalin (NGAL), a promising renal biomarker, can exists as a monomer, a dimer and/or in a NGAL/matrix metalloproteinase-9 (MMP-9) complex form when associated with different urinary diseases in humans and dogs. In this study, the presence of the various different molecular forms of NGAL in cat urine (uNGAL) was examined and whether these forms are correlated with different urinary diseases was explored.

**Results:**

One hundred and fifty-nine urine samples from cats with various different diseases, including acute kidney injury (AKI, 22 cats), chronic kidney disease (CKD, 55 cats), pyuria (44 cats) and other non-renal and non-pyuria diseases (non-RP, 26 cats), as well as healthy animals (12 cats), were collected. The molecular forms of and concentrations of urinary NGAL in these cats were analyzed, and their uNGAL-to-creatinine ratio (UNCR) were determined. The cats with AKI had the highest UNCR (median: 2.92 × 10^− 6^), which was followed by pyuria (median: 1.43 × 10^− 6^) and CKD (median: 0.56 × 10^− 6^); all of the above were significantly higher than the healthy controls (median: 0.17 × 10^− 6^) (*p* < 0.05). Three different NGAL molecular forms as well as the MMP-9 monomer were able to be detected in the cat urine samples. Moreover, the cases where urine NGAL monomer were present also had significantly higher levels of BUN (median: 18.9 vs 9.6 mmol/L) and creatinine (327.1 vs 168 umol/L). The presence of dimeric NGAL was found to be associated with urinary tract infections. Most cats in the present study (126/159, 79.2%) and more than half of healthy cats (7/12, 58.3%) had detectable NGAL/MMP-9 complex present in their urine.

**Conclusions:**

The monomeric and dimeric molecular forms of uNGAL suggest upper and lower urinary tract origins of disease, respectively, whereas the presence of the uNGAL/MMP-9 complex is able to be detected in most cats, including seemingly healthy ones.

## Background

Neutrophil gelatinase-associated lipocalin (NGAL), a 25 kDa glycoprotein belonging to lipocalin superfamily, was first purified from human neutrophils in the early 1990s [1, 2]. In humans and dogs, NGAL has been reported to serve as a biomarker for acute kidney injury (AKI) [3] and chronic kidney disease (CKD) [4–6]. In humans and dogs, NGAL is known exist in a number of forms, namely as 25 kDa monomers, as 45 kDa dimers, or in a 135 kDa NGAL/ MMP9 complex form where it is covalently conjugated with matrix metalloproteinase-9 (MMP9) [1, 7]. Compared to monomeric MMP-9 in urine, the urine NGAL/ MMP9 complex is characterized by having a higher molecular weight, and this conjugation with NGAL might help to protect MMP-9 from degradation [8]. Monomeric NGAL and, to some extent the dimeric form, has been reported to be the predominant forms produced by renal tubular epithelial cells, whereas dimeric NGAL is believed to be mainly secreted by neutrophils in humans [9].

In veterinary medicine, NGAL has been proved to be a novel and promising biomarker. An increased level of NGAL in dogs has been found to be associated with various different urinary diseases [5, 10–13]. Among cats with naturally occurring kidney diseases, urine NGAL (uNGAL) levels and the urine NGAL to creatinine ratio (UNCR) are thought to be a useful biomarkers when detecting chronic renal damage and when predicting the clinical progression of cats with CKD [14]. However, the presence and the various levels of the different molecular forms of uNGAL have not yet been investigated in cats. The first aim of this study was to determine the presence and origin of the various different molecular forms of uNGAL in cats. The second aim was to investigate the correlation in cats between the different molecular forms of uNGAL, as well as the concentration of uNGAL, with various different urinary diseases.

## Results

**Fig. 1:**
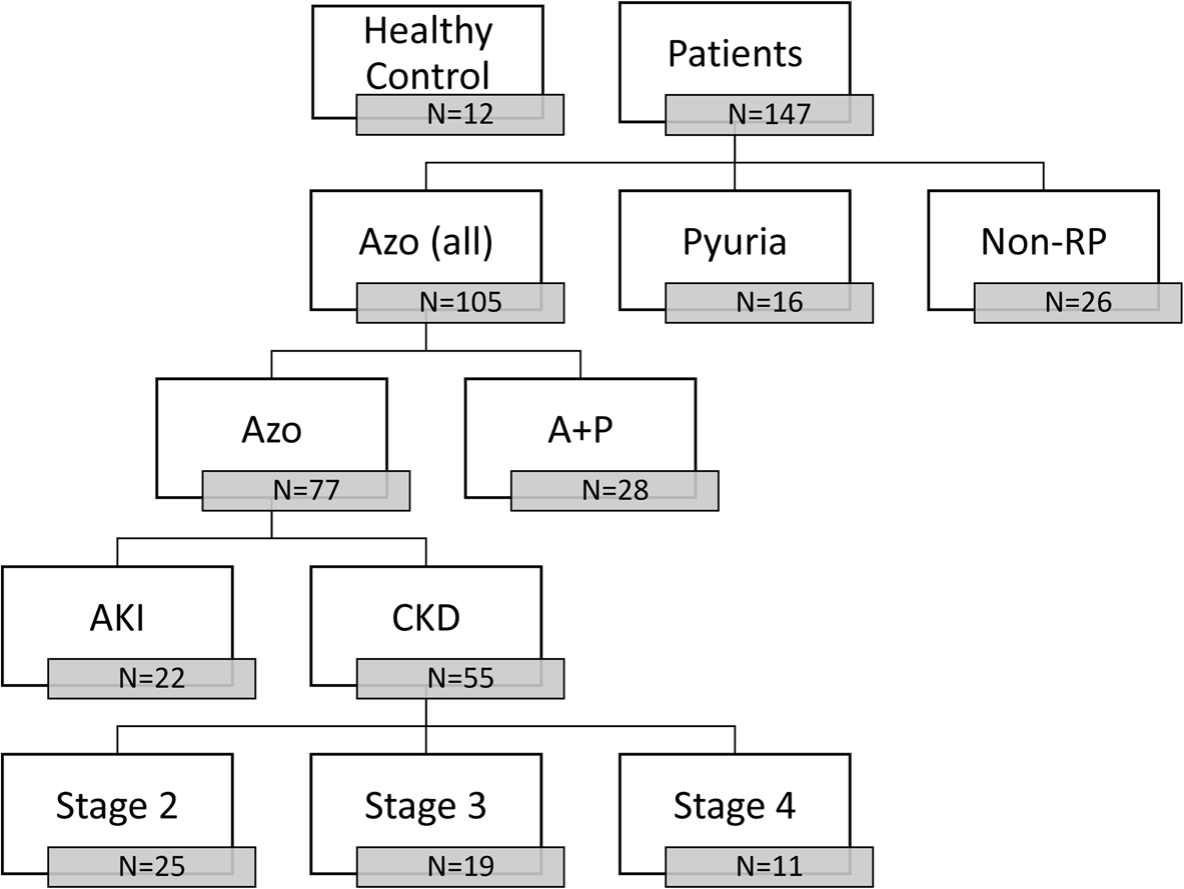
Diagram of the case grouping in this study. The Azo (all) group includes the cats with azotemia; the Azo group consists of the azotemia cats without pyuria, while the A + P group includes the cats having both pyuria and azotemia. The non-RP group consists of the cats with diseases unrelated to azotemia and pyuria; while the AKI group is made up of cats with acute kidney disease. The CKD group consists of cats with chronic kidney disease

In total, samples of 159 cats, which were able to be classified into a number of distinct groups (Fig. 1), were enrolled in the present study. The uNGAL concentrations were measured by ELISA.

The uNGAL concentration values and the UNCR of all of the groups are presented in Table 1**.** Urine samples from the A + P group had the highest level of uNGAL, which was followed by the AKI group, the pyuria group, the non-RP group, the healthy controls and finally the CKD group, in descending order. There were no significant differences in uNGAL levels among the A + P, AKI and pyuria groups. The uNGAL values of the A + P and AKI groups were significantly higher than those of the non-RP, control and CKD groups. The concentration of uNGAL obtained for the non-RP group was significantly higher than the values for the control and CKD groups. After adjusting the absolute value of uNGAL based on the urine creatinine concentration, the levels of UNCR were determined and compared among groups. The values for UNCR in descending order were the A + P group, the AKI group, the pyuria group, the CKD group, the non-RP group and the healthy control cats. The UNCR values of the A + P and AKI groups were significantly higher than the values for the CKD group, the non-RP group and the healthy controls. Significant differences in UNCR were found between the A + P group and the pyuria group, and between the AKI group and the CKD group, but no significant difference was found between the AKI group and the pyuria group.

**Table 1:** The differences in urinary NGAL and MMP-9 concentrations and various other parameters among the different groups

Parameter	Control		Non-RP		CKD		AKI		Pyuria		A + P		*p*
uNGAL (ng/ml)	0.46 (0.3–0.6) ^a^	*n =* 12	0.75 (0.5–0.9) ^b^	*n =* 26	0.37 (0.2–0.6) ^a^	*n =* 52	1.78 (0.8–6.6) ^c^	*n =* 21	1.21 (0.5–28.2) ^b, c^	*n =* 16	11.56 (1.1–47.5) ^c^	*n =* 27	< 0.001
UNCR (10^− 6^)	0.17 (0.1–0.3) ^a^	*n =* 12	0.37 (0.2–1.3) ^b^	*n =* 26	0.56 (0.2–1.3) ^b^	*n =* 52	2.92 (1.1–7.7) ^c, d^	*n =* 21	1.43 (0.4–26.2) ^c^	*n =* 16	5.50 (1.4–84.7) ^d^	*n =* 27	< 0.001
Age (years)	5.0 (3–6.8) ^a^	*n =* 12	7.5 (3.3–12.5) ^a^	*n =* 24	10.5 (7–15) ^b^	*n =* 52	11 (9–15) ^b^	*n =* 22	9.0 (4–11.8) ^a^	*n =* 16	8 (5.5–13.5) ^a^	*n =* 27	< 0.001
HCT (%)	40.9 (39–44.7) ^a^	*n =* 12	30.6 (27.5–36.1) ^b^	*n =* 23	31 (23–35.8) ^b^	*n =* 50	27.3 (22.2–33) ^b^	*n =* 21	30.5 (25.5–36) ^b^	*n =* 15	31 (24.6–38) ^b^	*n =* 28	< 0.001
BUN()	7.5 (7–8.8) ^a^	*n =* 12	7.1 (6.4–8.9) ^a^	*n =* 25	15.71 (10.7–23.4) ^b^	*n =* 55	54.4 (33.8–77.7) ^d^	*n =* 22	6.8 (4.9–8.3) ^a^	*n =* 15	20.2 (14.3–38.3) ^c^	*n =* 28	< 0.001
Cre (umol/L)	132 (123–132.6) ^b^	*n =* 12	110.5 (88.4–123.8) ^a^	*n =* 26	274.04 (221–406.7) ^c^	*n =* 55	1127.1 (495.2–1299.7) ^e^	*n =* 22	92.82 (70.4–123.8 ^a^	*n =* 16	393.38 (238.7–583.6) ^d^	*n =* 28	< 0.001
Phos (mmol/L)	–	–	1.45 (1.2–1.7) ^a, b^	*n =* 14	1.58 (1.3–2) ^b^	*n =* 49	3.68 (1.7–5.8) ^c^	*n =* 21	1.36 (0.81–1.49) ^a^	*n =* 9	2.2 (1.4–3.9) ^c^	*n =* 26	< 0.001
urine pH	6.4 (6–6.7) ^c, d^	*n =* 12	6.3 (6–6.6) ^c, d^	*n =* 26	6 (5.5–6.3)^b, c^	*n =* 55	5.6 (5.3–5.9) ^a^	*n =* 22	6.5 (6.2–7) ^d^	*n =* 16	6.1 (5.7–6.3) ^c^	*n =* 28	< 0.001
uRBC (/HPF)	0 (0–2) ^a^	*n =* 12	0 (0–12.5) ^a^	*n =* 26	0 (0–9) ^a^	*n =* 55	2.5 (0–9) ^a^	*n =* 22	55.5 (20.3–60) ^b^	*n =* 16	60 (18–60) ^b^	*n =* 28	< 0.001
uWBC (/HPF)	0 (0–0) ^a^	*n =* 12	0 (0–0) ^a^	*n =* 26	0 (0–1) ^a^	*n =* 55	1 (0–3.3) ^b^	*n =* 22	24 (9.5–53) ^c^	*n =* 16	60 (18.5–60) ^d^	*n =* 28	< 0.001

*CKD* Chronic kidney disease, *AKI* Acute kidney injury, *Pyuria* Pyuria without azotemia, *A + P* Azotemia with pyuria, *Non-RP* Diseases without any relation of azotemia and pyuria, *NGAL* Urinary NGAL, *UNCR* Urinary NGAL-to-creatinine ratio, *HCT* Hematocrit, *Cre* Creatinine, *Phos* Phosphate, *USG* Urine specific gravity, *uRBC* Urine RBC, *uWBC* Urine WBC, *HPF* High power field

Data are medians (IQR) and compared by the Kruskal-Wallis test (*p* < 0.05 indicates significant difference); Dunn’s test for the post hoc test and the different superscripts, ^a, b, c, d^ indicate various significant differences

**Fig. 2:**
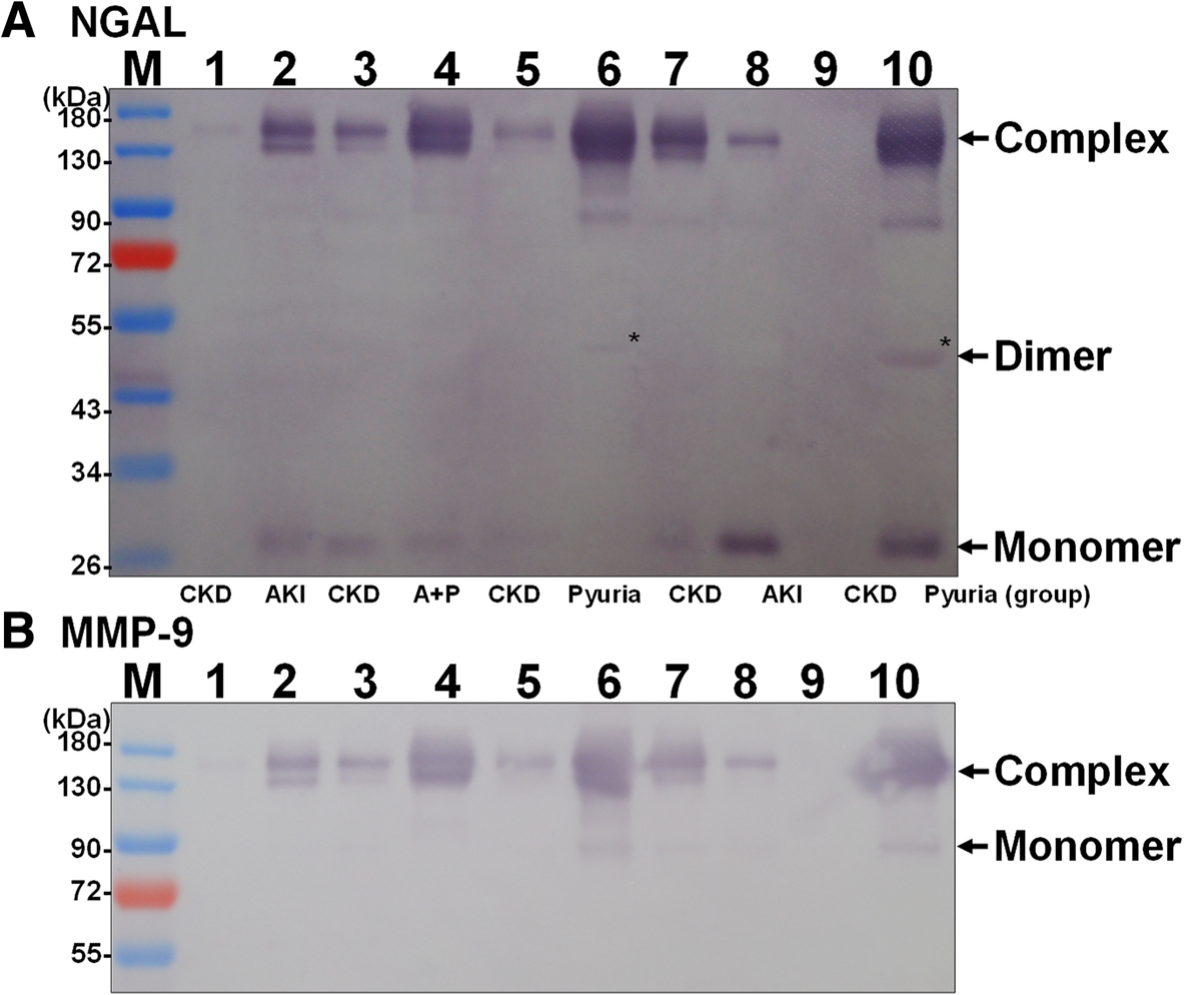
The molecular forms of NGAL and MMP 9 present in feline urine samples. The proteins in the urine samples from representative cats with CKD (lane 1, 3, 5, 7, 9), AKI (lane 2, 8), A + P (land 4), and pyuria (lane 6, 10) were separated by non-reducing SDS-PAGE. The presence of NGAL and MMP9 was detected by Western blot analysis using antibodies specific for NGAL (**a**) and MMP-9 (**b**). The protein complex with a molecular weight of 140-kDa was simultaneously detected by both antibodies, anti-NGAL (**a**) and anti-MMP-9 (**b**), and was therefore identified as the NGAL/MMP-9 complex

The molecular forms of the two target proteins in all of the enrolled urine samples were analyzed by Western blot analysis using rabbit anti-canine NGAL antibodies. Under non-reducing condition, three different molecular forms of uNGAL were successfully detected in the feline samples, namely the 27-kDa monomer, the 48-kDa dimer and the 140-kDa NGAL/MMP-9 complex (Fig. 2a). The existence of the 140-kDa NGAL/MMP-9 complex in feline urine was further confirmed by Western blot analysis using rabbit anti-canine MMP-9 antibodies. In addition, the 90-kDa MMP-9 monomer was also successfully identified in our feline urine samples (Fig. 2b).

Overall, uNGAL monomer was detected in 74 cats (48.1%, 74/154). Compared to the cats without uNGAL monomer, the cats with monomeric uNGAL on urinalysis were characterized as having a significantly higher level of uNGAL, a significantly higher level of UNCR, a lighter body weight, a lower level of HCT, a higher WBC count, a higher segmented neutrophil count, a higher BUN value, a higher creatinine value, a higher phosphorus in blood value, a higher level of UPC and a lower pH values (Table 2).

**Table 2:** The differences in various variables between the groups with or without uNGAL monomer present in the urine samples

Parameter	uNGAL Monomer (−) 52.2% (83/159)		uNGAL Monomer (+) 47.8% (76/159)		*p*
uNGAL (ng/ml)	0.5 (0.0.3–1.3)	*n =* 80	0.9 (0.5–2.9)	*n =* 74	0.006
UNCR (10^−6^)	0.4 (0.2–1.9)	*n =* 80	1.3 (0.5–4.8)	*n =* 74	< 0.001
Seg. (10^9/L)	6.1 (3.8–9.2)	*n =* 72	12.8 (6.8–20.8)	*n =* 75	< 0.001
BUN (mmol/L)	9.6 (7.5–16.4)	*n =* 81	18.92 (8.9–47.5)	*n =* 76	< 0.001
Creatinine (umol/L)	168 (132.6–282.9)	*n =* 83	327.1 (132.6–663.2)	*n =* 76	0.002
Phosphorus (mmol/L)	1.4 (1.2–2)	*n =* 56	1.9 (1.5–3.8)	*n =* 65	< 0.001
urine pH	6.2 (5.8–6.5)	*n =* 83	5.9 (5.5–6.3)	*n =* 76	< 0.001
UPC	0.1 (0–0.45)	*n =* 30	0.7 (0.4–1.9)	*n =* 29	< 0.001
uRBC (/HPF)	0 (0–21)	*n =* 83	2.5 (0–58)	*n =* 76	0.127
uWBC (/HPF)	0 (0–8)	*n =* 83	1 (0–9)	*n =* 76	0.240

*uNGAL* Urinary NGAL, *UNCR* Urinary NGAL-to-creatinine ratio, *WBC* White blood cells, *Seg*. Segmented neutrophil, *BUN* Blood urea nitrogen, *UPC* Urinary protein-to-creatinine ratio, *UWBC* Urine white blood cells, *URBC* Urine red blood cell, *HPF* High power field

Data are medians (IQR) and compared by the Mann-Whitney *U*-test (*p* < 0.05 indicates significant difference)

The presence of dimeric uNGAL was noted in 44 cats (27.7%, 44/159). The cats with dimeric uNGAL had significantly higher levels of uNGAL and UNCR. The significantly different parameters between the cats with and without dimeric uNGAL were a higher segmented neutrophil count in blood, a higher level of urinary protein, a higher urine RBC count and a higher urine WBC count. (Table 3).

**Table 3:** The differences in various variables between the group with or without NGAL dimer present in the urine

Parameter	uNGAL Dimer (−) 72.3% (115/159)		uNGAL Dimer (+) 27.7% (44/159)		*p*
uNGAL (ng/ml)	0.6 (0.3–1.2)	*n =* 111	2.3 (0.6–47.5)	*n =* 43	< 0.001
UNCR (10^−6^)	0.6 (0.2–1.9)	*n =* 111	3.4 (0.5–53.7)	*n =* 43	< 0.001
Seg. (10^9/L)	7.3 (4.3–14.3)	*n =* 106	11.1 (6.9–18.5)	*n =* 41	0.010
BUN (mg/dL)	33 (21–74)	*n =* 114	34 (21–78)	*n =* 43	0.937
Creatinine (mg/dL)	2.6 (1.5–5.6)	*n =* 115	2.2 (1.2–4.5)	*n =* 44	0.124
Phosphorus (mg/dL)	5.0 (4.2–8.9)	*n =* 86	5.1 (4.2–10)	*n =* 35	0.975
urine pH	6 (5.6–6.4)	*n =* 115	6.1 (5.7–6.4)	*n =* 44	0.543
UPC	0.3 (0.1–0.9)	*n =* 47	0.5 (0.1–1.7)	*n =* 12	0.491
URBC (/HPF)	0 (0–20)	*n =* 115	13 (0–60)	*n =* 44	< 0.001
UWBC (/HPF)	0 (0–2)	*n =* 115	14 (2–60)	*n =* 44	< 0.001

*uNGAL* Urinary NGAL, *UNCR* Urinary NGAL-to-creatinine ratio, *WBC* White blood cells, *Seg.* Segmented neutrophil, *BUN* Blood urea nitrogen, *UPC* Urinary protein-to-creatinine ratio, *UWBC* Urine white blood cells, *URBC* Urine red blood cell, *HPF* High power field

Data are medians (IQR) and compared by the Mann-Whitney *U*-test

In total, the NGAL/MMP-9 complex was present in urine of 126 cats (126/159, 79.2%). Furthermore, over half of the cats in healthy control group also had NGAL/MMP-9 complex present in their urine (7/12, 58.3%). No parameters were found to be significantly different between the cats with and without NGAL/MMP-9 complex across of the groups studied here.

Among the CKD group (*n =* 55), there were 25 cats in stage 2, 19 cats in stage 3 and 11 cats in stage 4. The occurrence frequencies of urinary monomeric NGAL, the NGAL/MMP-9 complex and monomeric MMP-9 were all in ascending order starting with the control group as lowest and increasing with stage. The proportion of cats with urinary NGAL monomer present was significantly higher for CKD stage 3 cats and CKD stage 4 cats compared to the control group cats and CKD stage 2 cats (both *p* < 0.001 for versus control group; both *p* < 0.05 versus CKD stage 2). (Table 4).

**Table 4:** The differences of urine NGAL and MMP-9 between the control and CKD subgroups

Parameter	Control (*n =* 12)	Stage 2 (*n =* 25)	Stage 3 (*n =* 19)	Stage 4 (*n =* 11)	*p*
uNGAL (ng/ml) ^d^	0.5 (0.3–0.6) ^a, b^ *n =* 12	0.3 (0.2–0.5) ^a^ *n =* 23	0.5 (0.3–0.9) ^b^ *n =* 18	0.6 (0.3–1.2) ^b^ *n =* 11	0.030*
UNCR (10^− 6^) ^d^	0.17 (0.1–0.3) ^a^ *n =* 12	0.17 (0.1–0.5) ^a^ *n =* 23	0.605 (0.3–1.4) ^b^ *n =* 18	1.33 (0.9–2.7) ^c^ *n =* 11	< 0.001**
NGAL Monomer ^e^	0% (0/12)	20% (5/25)	63.2% (12/19)	81.8% (9/11)	< 0.001**
NGAL Dimer ^e^	0% (0/12)	20% (5/25)	0% (0/19)	0% (0/11)	0.643
NGAL/MMP-9 ^e^	58.3% (7/12)	60% (15/25)	84.2% (16/19)	90.9% (10/11)	0.102

The different superscripts (^a, b, c^) indicate various significant differences

^d^Data are medians (IQR) and compared by the Kruskal-Wallis test (*p* < 0.05 indicated significant difference) and post hoc by Dunn’s test.

^e^Fisher’s exact test are used to compare the category data and presented in%.

*uNGAL* Urine NGAL, *UNCR* Urine NGAL-to-creatinine ratio

At the time of sample collection, 56 urine samples were subjected to the urine culture. According the results of these urine cultures, these 56 cats could be divided into a UTI (−) group (*n =* 38) and a UTI (+) group (*n =* 18). A comparison between these two groups indicated that the presence of dimeric NGAL was significantly higher in the cats with positive urine culture results (Table 5). Both uNGAL and UNCR were significantly correlated independently with the level of serum creatinine and the severity of pyuria (uWBC) (Table 6).

**Table 5:** The differences in various variables between UTI and non-UTI

Parameter	UTI (−) (*n =* 38)		UTI (+) (*n =* 18)		*p*
uNGAL (ng/ml) ^a^	0.8 (0.4–1.7)	*n =* 37	47.4 (6.1–48.2)	*n =* 18	< 0.001*
UNCR (10^−6^) ^a^	0.93 (0.5–2)	*n =* 37	41.77 (11–86)	*n =* 18	< 0.001*
NGAL Monomer (+) ^b^	60.5%	(23/38)	50. %	(9/18)	0.457
NGAL Dimer (+) ^c^	34.8%	(8/38)	83.3%	(15/18)	< 0.001*
NGAL/MMP-9 (+) ^b^	86.8%	(33/38)	83.3%	(15/18)	0.703

*UTI* Urinary tract infection

^a^ Data are medians (IQR) and tested by Mann-Whitney U test

^b^ Data are analyzed by the Chi-square test

^c^ Data are analyzed by the Fisher’s exact test

*p* < 0.05* indicates a significant difference

**Table 6:** The spearman’s correlations between uNGAL, UNCR, and plasma creatinine, urine WBC

Parameters	*r*	*p*
uNGAL and Creatinine	0.213	0.008
uNCR and Creatinine	0.406	< 0.001
uNGAL and uWBC	0.474	< 0.001
uNCR and uWBC	0.474	< 0.001

Creatinine: plasma creatinine, *uWBC* Urine WBC, *uNGAL* Urine NGAL *UNCR* Urine NGAL-to-creatinine ratio, r

## Discussion

To our knowledge, this is the first study to evaluate the presence of the different molecular forms of uNGAL in cats. The various molecular forms of uNGAL and MMP-9 in cats were able to be successfully detected by Western blot analysis using anti-canine NGAL and anti-canine MMP-9 antibodies. Similar to previous findings in human patients [9] and dogs [7], three different forms of NGAL were found to be present in the feline urine samples, these were the 27 kDa monomer, the 48 kDa dimer and the 140 kDa NGAL/MMP-9 complex forms. Additionally, the 90-kDa MMP-9 monomer was also successfully identified in our feline urine samples.

The results demonstrate that the appearance of NGAL monomer and MMP-9 monomer are both significantly associated with elevated levels of serum BUN, creatinine and phosphate, which are known to be linked with renal disease. These findings suggest that one or other of these or possibly both of these monomers may be associated with kidney injury in cats. Both uNGAL and UNCR have been reported to be promising renal biomarkers in cats with kidney disease [14]. Moreover, the UNCR of cats with azotemia is significantly higher than the values obtained from healthy control cats.

The comparison between the AKI and CKD groups revealed that cats with AKI have a significantly higher level of uNGAL and UNCR, as well as a higher proportion of NGAL monomer compared to CKD cats. These findings are similar to those presented in previous reports targeting humans [15] and dogs [10]. The NGAL monomer has been considered to originate from injured renal tubular epithelial cells [9]. Therefore, the higher proportion of cats with NGAL monomer in the AKI group than in the CKD group seems to reflect the severity of the renal tubular injury during feline AKI. However, the influence of glomerular or renal hemodynamic dysfunction cannot be accessed and further investigation was needed.

Surprisingly, the NGAL/MMP-9 complex was found to be present in the urine of the majority of cats enrolled in the present study (126/159, 79.2%). This included more than half of the cats in the healthy control group (7/12, 58.3%). This finding is different from previous findings in dogs [7] and humans [9]. The presence of the NGAL/MMP-9 complex in the urine of normal cats may indicate that the NGAL/MMP-9 complex may play a role in the normal physiology of the cat urinary system or, on the other hand, that the healthy control cats had already some abnormalities that could not be detected by the traditional examination. This area is well worth further investigation.

In dogs, the presence of WBCs in urine is correlated with an increase in urine NGAL level [7, 16]. Additionally, the presence of dimeric uNGAL has been found to be related to WBC counts in canine urine samples [7]. Consistently, the results of the present study reveal that the uNGAL and UNCR of cats in the pyuria group are both significantly higher than of the values for the healthy control cats. Moreover, the presence of dimeric NGAL was found to be significantly associated with the urine WBC counts in cats. The proportion of cats with dimeric uNGAL is significantly higher in the pyuria group than in the healthy control group. In this study, the pyuria group consists of cats without azotemia, but with other diseases. These findings indirectly demonstrate that the feline monomer uNGAL seems to originate from the kidneys, and feline dimeric uNGAL appears to originate from inflammation of lower urinary tract. Increased uNGAL has been reported to be associated with neoplasia and endotoxaemia [13]. In this study, monomeric NGAL is the predominant molecular form in the urine of the cats that form the non-RP group (data not shown). However, the renal function of the cats in the non-RP group was only evaluated using BUN, creatinine and urinary analysis, and thus early injury to the kidneys cannot be excluded. Additionally, various neoplastic diseases and/or septic illnesses also cannot be ruled out.

The proportion of cats with uNGAL monomer and concentrations of uNGAL and UNCR that were significantly higher were only present in cats with either CKD IRIS stage 3 or stage 4, as compared to either the control group or CKD IRIS stage 2 group. These results suggest that these variables may be useful as progression factors [14] in relation to late stage CKD.

When the different molecular forms of these proteins were compared between cats with and without UTI, the results indicated that dimeric uNGAL exists as the predominate molecular form in the urine of cats with UTI. This finding suggests that the presence of UTI should be considered when interpreting urine NGAL values when feline renal disease is present. Moreover, it is likely the dimeric uNGAL might be derived from neutrophils [9], and thus might play a role in diseases where bacteria are present in feline urine.

There are several limitations in this study. First, urine bacterial culture should be the gold standard when diagnosing UTI; nevertheless not all of the cats in the present study underwent urine bacterial culture and this means that the UTI case numbers might be an underestimate. Under such circumstances, any elevation of uNGAL might be with the result of an undetected UTI. Second, feline idiopathic cystitis is thought to be an important lower urinary disease that involves cystic inflammation, but the design of this study means that we were unable to investigate the role of dimeric uNGAL in this disease. Third, although we tried to group the cases in a simple manner, the cats from which the clinical samples were obtained may in some cases have complicated urinary diseases. This means that our study does not provide an animal model approach that would be able to explore directly the role of each specific uNGAL molecular form in a given disease.

## Conclusions

To date, an ELISA kit that allows the differential detection of the various molecular forms of NGAL is not available. To comprehensively understand the presence of these NGAL forms in feline urine, Western blot analysis remains the ideal system for the simultaneous differentiation of the various molecular forms of uNGAL based on their molecular weights. As with humans and dogs, the presence of the uNGAL monomer in cats seems to be correlated with renal injury, whereas the presence of dimeric uNGAL appears to be involved in pyuria and UTI. Nevertheless, unlike humans and dogs, the uNGAL/MMP-9 complex is able to be detected in most cats, even healthy ones.

## Methods

### Cats and sample collection

Feline urine samples were prospectively obtained from the cats that were admitted to National Taiwan University Veterinary Hospital, Taipei, Taiwan as part of feline routine diagnosis (IACUC Approval No: NTU 103-EL-00084). Most feline urine samples were collected by cystocentesis (73.6%, 117/159). However, samples were also obtained by catheterization (5.7%, 9/159), voiding (15.1%, 24/159), or aspiration using a subcutaneous ureteral bypass (SUB) device (1.3%, 2/159) and these samples were also enrolled. Finally, there were seven urine samples (4.4%, 7/159) that had an unrecorded urine collection method. The fresh urine samples were clarified by centrifugation at 1500 rpm for 5 min; the time between collection and centrifugation was limited within 30 min. The urine supernatants were stored in microcentrifuge tubes at − 80 °C until NGAL analysis. Important clinical information about each case, including history, the results of any physical examination and the results related to hematology, serum/plasma biochemistry and urinalysis were recorded for each cat. These parameters, including the number of segmented neutrophils in the blood, serum BUN, creatinine, phosphorus and urine protein-to-creatinine ratio (UPC), uWBC, and uRBC, all of which have been reported to be associated with uNGAL, were used for the comparisons between the groups with and without the specific molecular forms of uNGAL. Blood chemistry, the urine protein/creatinine ratios and the complete blood counts were measured using a Vitros 350 chemistry system (Ortho clinical diagnostics), an IDEXX Catalyst Dx* Analyzer (IDEXX laboratories, Inc.), and an Exigo, (Boul Medical AB) respectively.

### Case grouping criteria

The urine samples were classified into a number of different groups (Fig. 1). Cats without any clinical signs associated with any disease, with unremarkable physical examination findings and that had normal values for hematocrit (HCT), white blood cells (WBC), segmented neutrophils, alanine aminotransferase, aspartate transaminase, alkaline phosphatase, albumin, total protein, glucose, blood urea nitrogen (BUN), and creatinine, as well as the absence of any abnormalities on urinalysis, were classified as healthy control animals (BUN < 10.7 mmol/L, creatinine< 140 umol/L, WBC in urine sediment< 5 /high power field (HPF). The healthy cats were mainly cats belonging to students and colleagues, although some were cats that had been brought in to be neutered by surgery.

Cats with azotemia (creatinine > 140 umol/L), and with a history and clinical signs that supported renal disease, were enrolled into the azotemia group. Based on the results of urinalysis, these azotemic cats were further divided into an azotemia-pyuria group (with > 5 WBCs/HPF in sediment) and an azotemia group. However, cats with prerenal azotemia, where the azotemia was resolved after fluid therapy within 24 h, were excluded.

The cats with persistent azotemia (creatinine > 140 umol/L longer than 1 month of duration) and with one of the clinical findings associated with CKD (e.g. polyuria, polydipsia, urine specific gravity < 1.030, one or more small irregular kidneys (defined as one having a renal size of less than two times the length of the second lumber vertebrae by ventral dorsal view radiography or with decreased corticomedullary distinction by ultrasonography)) were included in the CKD group. The azotemia-CKD group was further classified into three subgroups based on the International Renal Interest Society (IRIS) staging system, these were (a) IRIS CKD stage II, (b) IRIS CKD stage III, (c) IRIS CKD stage IV. Cats with an acute onset of clinical signs shorter than 7 days (including those with oliguria, anuria, glucosuria, urinary cast, and enlarged kidneys), or with a plasma creatinine concentration increase of ≥26.5 umol/L from baseline within 48 h, were defined as the AKI group. The cats without azotemia (creatinine < 140 umol/L) and with > 5 WBCs/HPF on urine sediment examination were categorized into the pyuria group.

Cats with a normal plasma creatinine concentration (values < 140 umol/L), and with < 5 WBCs/HPF in urine sediment, but with other diseases, were classified into the non-renal and non-pyuria (non-RP) group; this consisted of eight cases with neurological diseases, three cases with an infectious disease, three cases with a hepatic disease, two cases with a gastrointestinal disease, one case of aortic thromboembolism, one case of aplastic anemia, one case of pyometra and mammary gland tumor, one case of pyoperitoneum, one case of diabetes mellitus, one case of pancreatitis, one case of mast cell tumor, one case of hiatal hernia, one case of vaccine-associated sarcoma, and one case of lingual disease.

### Western blot analysis

Urine samples were 4:1 diluted with premixed Laemmli protein sample buffer (4x Laemmli Sample Buffer, Bio-Rad Laboratories Inc.) before the proteins were separated by non-reducing sodium dodecyl sulfate polyacrylamide gel electrophoresis (SDS-PAGE). The protein mix was directly loaded onto the gel without boiling. Subsequently, after SDS-PAGE, the gels were electrophoretically transferred to polyvinylidene difluoride (PVDF) membranes. The Western blot analysis followed the procedures that were described in a previous report [7]. The sequences of the dog and cat MMP-9 s (Dog: NM_001003219.2; cat; XM_0039834612.4) shares 93% similarity and therefore initially both canine and feline MMP-9 proteins were simultaneously detected by antibody against canine MMP-9 [7]. This confirmed that the canine-MMP-9 antibody was able to bind to MMP-9 proteins from both species; for that point onwards, canine-MMP-9 antibody was usedto detect the feline protein.

Briefly, for Western blotting, the membrane was blocked with phosphate buffered saline (PBS) containing 0.1% Tween-20 (PBST) and 5% dried skimmed milk for 1 h at room temperature and this was followed by incubation with a 1000-fold dilution of rabbit anti-canine antibodies against NGAL or MMP-9 overnight at 4 °C. After washing with PBST three times, the membrane was incubated with a 10,000-fold dilution of secondary alkaline phosphatase (AP) conjugated anti-rabbit antibody for 1 h at room temperature. After further washes with PBST to remove the unbound antibodies, the signal on membrane was developed using 5-bromo-4-chloro-3-indolyl phosphate (BCIP)/nitro blue tetrazolium (NBT) solutions (AP Conjugate Substrate Kit, Bio-Rad Laboratories Inc.) in the dark for 15 min, and the image was recorded by digital camera.

Based on the expression pattern of the molecular forms of NGAL and MMP-9, all the cases were classified into the following categories: NGAL monomer (+) and NGAL monomer (−) groups; NGAL dimer (+) and NGAL dimer (−) groups; and NGAL/MMP-9 complex (+) and NGAL/MMP-9 complex (−) groups.

### Measurement of urine NGAL concentrations by ELISA

Sandwich enzyme-linked immunosorbent assays (ELISAs) were conducted using the procedures described in a previous study [14]. Briefly, the capture anti-NGAL mouse polyclonal antibody was diluted 1: 800 fold in coating buffer and then was added to each well of an ELISA plate (Nunc TM). After blocking, 2-fold diluted urine samples and serially diluted feline recombinant NGAL calibration samples (with concentrations ranging from 17 ng/mL to 17 pg/mL and 0 pg/mL) were individually added to each well and incubated at 4 °C for 16 h. Each experiment was carried out in duplicate. After washing with PBST three times, each well was loaded with 100 μl of 2000-fold diluted anti-NGAL rabbit polyclonal antibodies for detection and this was followed by incubation at 37 °C for 2.5 h. Subsequently, a 10,000-fold dilution of horseradish peroxidase (HRP) conjugated anti-rabbit secondary antibody was added to each well. After 1 h incubation, the plates were washed and TMB substrate (Tetramethylbenzidine, Clinical Science Laboratory, Inc.) was added, followed by incubation of the plate in the dark for 15 min. The reaction was terminated by adding 2 N H_2_SO_4_ and then the OD value was measured at 450 nm wavelength using a microplate ELISA reader (TECAN). The results of the duplicate experiments were averaged and the NGAL levels are expressed as nanograms per milliliter (ng/ml).

The precision and repeatability of the ELISA were evaluated by the coefficient of variation (CV) intra-assay and inter-assay analysis using three concentrations as calibrators (8625, 1078.1, 33.69 pg/mL; these were indicated as high, medium and low concentrations of the protein). The coefficient of variation (CV) of the average intra-assay and inter-assay CVs were 6.94 and 5.76%, respectively. Additionally, based on the standard curve established by calibrators (standard curve with R^2^ = 0.99847), NGAL protein levels in clinic samples were measured. All of the ELISAs for measuring NGAL involved three independent repeats. The cut-off value for detection was set by means of negative protein at + 3 standard deviations (SD). In general, the detection limit of in-house ELISA was estimated based on the lowest concentration of calibrator with a read higher than cutoff value.

### Statistical analysis

Statistical Product and Service Solutions (SPSS® 20 for Mac) statistical software was used for analysis in this study. Data were first analyzed using the Shapiro-Wilk test to determine the normality. Non-normally distributed data are presented as median and interquartile range (IQR). The Mann-Whitney *U*-test and Kruskal-Wallis Test (post hoc test with Dunn’s test) were utilized for the nonparametric analysis to compare the differences between groups or among two groups, respectively. Categorical data are presented as proportions. The chi-square test or Fisher’s Exact Test (for any expected cell number < 5) were used to compare these datasets. A *p value p* < 0.05 was considered to indicate a significant difference. Spearman’s correlation coefficients were applied to determine the associations between different parameters.

## Data Availability

The datasets used and/or analysed during the current study are available from the corresponding author on reasonable request.

## Abbreviations

AKI: Acute kidney disease
ANOVA: Analysis of variance
BCIP: 5-bromo-4-chloro-3-indolyl phosphate
CKD: Chronic kidney disease
ELISA: Enzyme-linked immunosorbent assay
HPF: High power field
IQR: Interquartile ranges
LSD: Least significant difference
MMP-9: Matrix metallopeptidase 9
NBT: Nitro blue tetrazolium
NGAL: Neutrophil gelatinase-associated lipocalin
PBST: Phosphate buffered saline containing 0.1% Tween-20
RBC: Red blood cell
ROC: Receiver operating characteristics
SDS-PAGE: Sodium dodecyl sulfate polyacrylamide gel electrophoresis
UNCR: Urine NGAL to creatinine ratio
UPC: Urine protein to creatinine ratio
UTI: Urinary tract inflammation
WBC: White blood cell count
